# Prior infection by seasonal coronaviruses, as assessed by serology, does not prevent SARS-CoV-2 infection and disease in children, France, April to June 2020

**DOI:** 10.2807/1560-7917.ES.2021.26.13.2001782

**Published:** 2021-04-01

**Authors:** Isabelle Sermet-Gaudelus, Sarah Temmam, Christèle Huon, Sylvie Behillil, Vincent Gajdos, Thomas Bigot, Thibaut Lurier, Delphine Chrétien, Marija Backovic, Agnès Delaunay-Moisan, Flora Donati, Mélanie Albert, Elsa Foucaud, Bettina Mesplées, Grégoire Benoist, Albert Faye, Marc Duval-Arnould, Célia Cretolle, Marina Charbit, Mélodie Aubart, Johanne Auriau, Mathie Lorrot, Dulanjalee Kariyawasam, Laura Fertitta, Gilles Orliaguet, Bénédicte Pigneur, Brigitte Bader-Meunier, Coralie Briand, Vincent Enouf, Julie Toubiana, Tiffany Guilleminot, Sylvie van der Werf, Marianne Leruez-Ville, Marc Eloit

**Affiliations:** 1 Institut Necker Enfants Malades, INSERM U 1171, Paris, France; 2 Hôpital Necker-Enfants Malades, Assistance Publique Hôpitaux de Paris, Paris, France; 3 Université de Paris, Paris, France; 4 These authors contributed equally to the work; 5 Pathogen Discovery Laboratory, Department of Virology, Institut Pasteur, Paris, France; 6 Molecular Genetics of RNA Viruses, Department of Virology, CNRS UMR3569, Université de Paris, Institut Pasteur, Paris, France; 7 National Reference Center for Respiratory Viruses, Institut Pasteur, Paris, France; 8 Hôpital Antoine Beclere, Clamart, France; 9 Centre for Research in Epidemiology and Population Health, INSERM UMR1018, Villejuif, France; 10 Hub de Bioinformatique et Biostatistique – Département Biologie Computationnelle, Institut Pasteur, USR 3756 CNRS, Paris, France; 11 Université Clermont Auvergne, INRAE, VetAgro Sup, UMR EPIA, Saint-Genès-Champanelle, France; 12 Université de Lyon, INRAE, VetAgro Sup, UMR EPIA, Marcy l’Etoile, France; 13 Université de Lyon, INRAE, VetAgro Sup, Usc 1233 UR RS2GP, Marcy l’Etoile, France; 14 Unité de Virologie Structurale, Institut Pasteur, Département de Virologie, CNRS, UMR3569, Paris, France; 15 Université Paris-Saclay, CEA, CNRS, Institute for Integrative Biology of the Cell (I2BC), Gif-sur-Yvette, France; 16 Hôpital Jean Verdier, Bondy, France; 17 Hôpital Louis Mourier, Colombes, France; 18 Hôpital Ambroise Paré, Boulogne Billancourt, France; 19 Hôpital Robert Debré, Paris, France; 20 Hôpital Kremlin Bicêtre, Le Kremlin-Bicêtre, France; 21 Hôpital Armand Trousseau, Paris, France; 22 Plateforme de microbiologie mutualisée (P2M), Pasteur International Bioresources Network (PIBnet), Institut Pasteur, Paris, France; 23 Unité Biodiversité et Epidemiologie des Bacteries Pathogènes, Institut Pasteur, Paris, France; 24 Laboratoire de Microbiologie, Hôpital Necker-Enfants Malades, Paris, France; 25 Ecole Nationale Vétérinaire d’Alfort, Maisons Alfort, France

**Keywords:** SARS-CoV-2, COVID-19, NL63, 229E, OC43, HKU1

## Abstract

**Background:**

Children have a low rate of COVID-19 and secondary severe multisystem inflammatory syndrome (MIS) but present a high prevalence of symptomatic seasonal coronavirus infections.

**Aim:**

We tested if prior infections by seasonal coronaviruses (HCoV) NL63, HKU1, 229E or OC43 as assessed by serology, provide cross-protective immunity against SARS-CoV-2 infection.

**Methods:**

We set a cross-sectional observational multicentric study in pauci- or asymptomatic children hospitalised in Paris during the first wave for reasons other than COVID (hospitalised children (HOS), n = 739) plus children presenting with MIS (n = 36). SARS-CoV-2 antibodies directed against the nucleoprotein (N) and S1 and S2 domains of the spike (S) proteins were monitored by an in-house luciferase immunoprecipitation system assay. We randomly selected 69 SARS-CoV-2-seropositive patients (including 15 with MIS) and 115 matched SARS-CoV-2-seronegative patients (controls (CTL)). We measured antibodies against SARS-CoV-2 and HCoV as evidence for prior corresponding infections and assessed if SARS-CoV-2 prevalence of infection and levels of antibody responses were shaped by prior seasonal coronavirus infections.

**Results:**

Prevalence of HCoV infections were similar in HOS, MIS and CTL groups. Antibody levels against HCoV were not significantly different in the three groups and were not related to the level of SARS-CoV-2 antibodies in the HOS and MIS groups. SARS-CoV-2 antibody profiles were different between HOS and MIS children.

**Conclusion:**

Prior infection by seasonal coronaviruses, as assessed by serology, does not interfere with SARS-CoV-2 infection and related MIS in children.

## Introduction

Coronavirus disease (COVID-19) is caused by infection with severe acute respiratory coronavirus 2 (SARS-CoV-2), a betacoronavirus of the subgenus *Sarbecovirus* [1], which has expanded worldwide since its emergence in China at the end of 2019. Observations indicate that children are less likely to develop the disease and that the clinical course of COVID-19 in children is less severe than in adults, but the reason why is still unknown [2-4]. Children represent only 0.6–2.3% of confirmed cases in China and 0.8–5.2% outside China, excluding household contacts [2,5,6]. As asymptomatic or mildly symptomatic children are underdiagnosed and their viral loads are comparable to those of adults, it is still uncertain whether children may act as an asymptomatic reservoir for the spread of the virus to their adult and elderly relatives [7,8], albeit with low efficacy [9-13]. It has also been suggested that children’s susceptibility to infection might be low [5]. This might be related to infections with seasonal human coronaviruses (HCoV) which are frequent at a very young age and result in mild respiratory infections [14,15]. They could lead to cross-protective immunity in children, mediated either by cross-binding or cross-neutralising antibodies [16] or by T-cell responses that target epitopes shared by SARS-CoV-2 and HCoV [17,18]. Indeed, it has recently been shown that CD4^+^ T-cells of unexposed subjects (sampled before the pandemic) recognised SARS-CoV-2 [17].

Cases of multisystem inflammatory syndrome (MIS) have been reported in children that were infected by SARS-CoV-2 or were in contact with COVID-19 patients [19,20]. As for seasonal coronaviruses [21], it is possible that a low antibody response to SARS-CoV-2 or cross-reactive antibodies facilitate immune-dependent enhancement following re-exposure, potentiated by a specific genetic background [22,23]. Interestingly, a domain of the SARS-CoV-2 spike protein which binds with high affinity to T-cells may act as a super antigen and trigger excessive adaptive immune responses [24].

The aim of this study was to analyse the impact of endemic seasonal coronavirus infection on SARS-CoV-2 infection in children by investigating in depth the typology of respective humoral responses, based on a luciferase immunoprecipitation system (LIPS) assay targeting the spike (S) and the nucleoprotein (N) of SARS-CoV-2 [22] and the four seasonal coronaviruses. We measured if prior infections with HCoV, evidenced by antibody responses, modulate the risk of SARS-CoV-2 infection by analysing the frequency and the level of response in SARS-CoV-2-positive children as compared with SARS-CoV-2-negative matched controls. We also analysed humoral responses against SARS-CoV-2 and seasonal HCoV in patients with MIS regarding antibody targets.

## Methods

### Cohort design 

Paediatric patients aged 0–18 years consulting or hospitalised for any disease other than COVID-19 for at most 4 days in paediatric tertiary healthcare departments of the Assistance Publique-Hôpitaux de Paris between 1 April and 1 June 2020 were included in an ongoing prospective multicentric observational seroprevalence study. We considered all patients presenting with a MIS disease, as defined by the American Heart Association [25]. 

To detect previous SARS-CoV-2 infection, we used an in-house LIPS assay targeting domain S1 of the S protein and the C-terminal part of the N protein as first line, as previously described [26]. The overall sensitivity of the LIPS assay was further improved by including the detection of antibodies against the S2 subdomain (Supplement). We identified three sub-cohorts of 54 SARS-CoV-2-seropositive hospitalised children (HOS-P), 15 SARS-CoV-2-seropositive children with MIS (MIS-P) and 115 SARS-CoV-2-seronegative children as controls (CTL), matched for their age and sex.

### Serological assays

To measure if prior infections with HCoV could influence the SARS-CoV-2 antibody response, the LIPS assays were extended to detect additional antibodies directed against the full S ectodomain (in a pre-fusion conformation) of SARS-CoV-2, the two human betacoronaviruses (HKU1 and OC43) and one human alphacoronavirus (229E). Assays similar to SARS-CoV-2 LIPS-N were also designed for the four HCoV, including the alphacoronavirus NL63. Detailed technical information is given in Supplement part 1. The sensitivity and specificity of the first line SARS-CoV-2 LIPS test (defined as a positive detection for either S1, S2 or N antigen) used to include the patients was 88% and 95.6%, respectively. Sensitivity and specificity calculation of all assays are detailed in Supplement part 2. 

### Statistical analysis

Statistical analyses were conducted with GraphPad Prism 8 (GraphPad Software, San Diego). The signal-to-noise light unit (LU) ratios between the three groups of children were compared using the Kruskal-Wallis ANOVA and Dunn’s multiple comparisons tests for each antigen considered. Significant differences of seroprevalence between groups were calculated using Fisher’s exact test. Two-sided p value < 0.05 was considered as significant. Principal component analysis (PCA) was performed to identify the serological profile according to SARS-CoV-2 antibodies and seasonal HCoV antibodies. Data were processed with R 3.6.3 [27] using GGPlot2 with GGally for matrices of plots, and ggfortify for PCA plots packages.

### Ethical statement

The local Ethics committee (CERAPHP Paris V) approved this study (IRB registration: #00011928). Serology was sampled for usual care and patients and/or their parents/guardians were informed about the study but did not have to provide consent, according the French legislation. 

## Results

### Patients

Table 1 presents the demographic and clinical characteristics of 54 SARS-CoV-2-seropositive HOS-P children, 15 SARS-CoV-2-seropositive MIS-P children and 115 SARS-CoV-2-seronegative CTL children. The comparison between HOS-P and CTL did not show any significant differences for age, sex ratio or reasons for hospitalisation.

**Table 1:** Demographic and clinical characteristics of the different groups of SARS-CoV-2-seropositive and -seronegative children, France, April–June 2020 (n =184)

	HOS-P SARS-CoV-2-positive n = 54		MIS-P SARS-CoV-2-positive n = 15		CTL SARS-CoV-2-negative n = 115		p (HOS-P vs CTL)	p (MIS-P vs CTL)	p (HOS-P vs MIS-P)
	n	%	n	%	n	%			
Demographic characteristics									
Age in years: mean (SD) (min–max)	9.8 (5.5) (0–18)		8.6 (3.4) (3–14)		9.6 (5.2) (0–18)		NS	NS	NS
Male sex	25	46	5	33	63	55	NS	NS	NS
Reason for hospitalisation									
Systematic monitoring	30	56	0	0	61	53	NS	< 10–4	< 10–4
Paediatric emergency	6	11	14	93	7	6	NS	< 10–6	< 10–6
Surgery	6	11	0	0	16	14	NS	NS	NS
Chronic disease exacerbation	2	4	0	0	0	0	NS	NS	NS
Comorbidities									
No comorbidity	32	59	13	87	63	55	NS	0.02	< 10–4
Diabetes	2	4	0	0	4	3	NS	NS	NS
Immunosuppression	8	15	0	0	8	7	NS	NS	0.005
Cancer in the 3 previous years	6	11	0	0	3	3	0.02	NS	NS
Others	22	41	2	13	37	32	NS	NS	NS
History consistent with COVID-19 in the 3 previous months ^a^									
Case contacts	8	24	2	26	10	9	0.006	NS	NS
Delay known exposure/sampling: mean (SD) (min–max)	39.6 (14.6) (32-71)		29 (1.4) (28–30)		NA		NA	NA	NA
Delay symptom onset/sampling: mean (SD) (min–max)	NA		29.9 (16.7) (7–64)		NA		NA	NA	NA
No symptoms	34	63	12	80	79	69	NS	NS	NS
Fever	14	26	0	0	13	11	0.01	NS	0.04
Diarrhoea, abdominal pain, vomiting	9	17	1	7	16	14	NS	NS	NS
Asthenia	7	13	1	7	6	5	0.03	NS	NS
Cough	2	4	0	0	6	5	NS	NS	NS
Dyspnoea/shortness of breath	2	4	1	7	1	1	NS	NS	NS
Headache	5	9	0	0	3	3	NS	NS	NS
Neuromuscular disorders	7	13	1	7	5	4.3	0.03	NS	NS
Rhinopharyngitis	5	9.2	0	0	5	4.3	NS	NS	NS
Dermatological symptoms	4	7.4	0	0	5	4.3	NS	NS	NS

COVID-19: coronavirus disease; CTL: SARS-CoV-2-seronegative control group; HOS-P: SARS-CoV-2-seropositive hospitalised patients who did not develop an MIS; MIS-P: SARS-CoV-2-seropositive patients with multisystemic inflammatory syndrome; NA: not available; NS: non-significant; SARS-CoV-2: severe acute respiratory syndrome coronavirus 2; SD: standard deviation.

**^a^** More than one answer was possible .

Data as mean (SD) or %.

Sixty-three per cent of the seropositive patients did not report any history consistent with COVID-19 during the preceding weeks. Among those, the MIS-P did not report any COVID-19 symptoms. The only symptoms that were marginally but significantly reported in the previous months in seropositive children were fever, asthenia, and myalgia. MIS-P patients did not report underlying chronic diseases and were all hospitalised in emergency units. There was no case of COVID-19 symptoms recorded before the onset of MIS and MIS-P patients were sampled at the time of MIS symptom onset. The mean delay between initial symptom onset and the time of serum sampling for MIS-P was 29.9 days (standard deviation (SD): 16.7 days; range: 7–64 days) (Table 1). Seropositive patients (MIS-P and HOS-P) reported significantly more case contacts in the 3 previous months. In total, 10 seropositive patients acknowledged known exposure with a mean delay of 37.5 days (SD: 14.4 days; range: 28–71 days). Two of 15 MIS-P patients were confirmed by positive SARS-CoV-2 RT-PCR at the time of hospitalisation for MIS. For the 13 remaining patients, two reported a known exposure, with a delay of 28 and 30 days, respectively. We cannot exclude another viral infection for these 15 patients, except that they were negative at the time of hospitalisation for human respiratory syncytial viruses, seasonal coronaviruses, parainfluenza and influenza viruses, metapneumovirus and rhinovirus/enterovirus in nasopharyngeal swabs.

### Immunoprofiling of SARS-CoV-2 infection in HOS-P and MIS-P children

**Figure 1:**
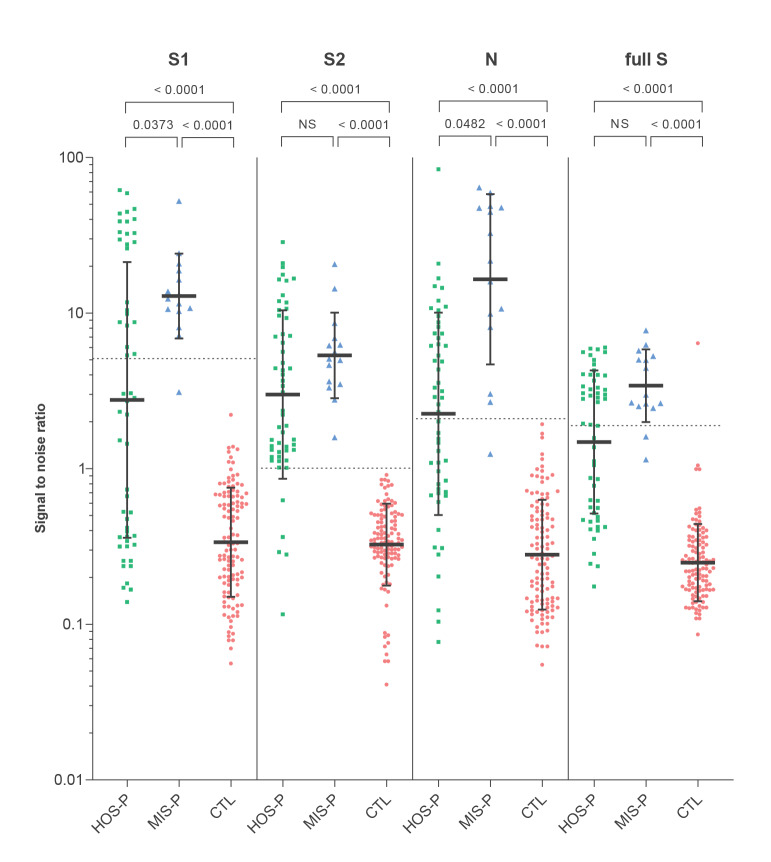
SARS-CoV-2 antibody responses in HOS-P, MIS-P and CTL children, France, April–June 2020 (n =184)

CTL: SARS-CoV-2-seronegative control group; HOS-P: SARS-CoV-2-seropositive hospitalised patients who did not develop an MIS; MIS-P: SARS-CoV-2-seropositive patients with multisystemic inflammatory syndrome; NS: non-significant; SARS-CoV-2: severe acute respiratory syndrome coronavirus 2.

The prevalence of children seropositive for SARS-CoV-2 S1, S2 or N differed between HOS-P and MIS-P children (Table 2). HOS-P patients were characterised by a dominant S2 response compared with responses to S1, N and to the full S ectodomain, whereas MIS-P patients’ antibodies were directed against N, S1 and S2 altogether. The profile of antibody responses to SARS-CoV-2 in seropositive HOS-P and MIS-P patients is shown in Figure 1. Levels of SARS-CoV-2 antibodies against N and S1 were significantly higher in the MIS-P than in the HOS-P group.

**Table 2:** Prevalence of antibodies to SARS-CoV-2 spike (full S ectodomain, S1 and S2 domains) and nucleoprotein in HOS-P, MIS-P and CTL children, France, April–June 2020 (n =184)

Antigen	HOS-P SARS-CoV-2-positive n = 54		MIS-P SARS-CoV-2-positive n = 15		CTL SARS-CoV-2-negative n = 115		p (CTL vs HOS-P)	p (CTL vs MIS-P)	p (HOS-P vs MIS-P)
	n	%	n	%	n	%			
S1	24	44.4	14	93.3	0	0.0	< 0.0001	< 0.0001	0.0008
S2	49	90.7	15	100	0	0.0	< 0.0001	< 0.0001	NS
N	29	53.7	14	93.3	0	0.0	< 0.0001	< 0.0001	0.0058
S1 or S2 or N	54	100	15	100	0	0.0	< 0.0001	< 0.0001	NS
Full S	28	51.9	13	86.7	1	0.9	< 0.0001	< 0.0001	0.0181

CTL: SARS-CoV-2-seronegative control group; HOS-P: SARS-CoV-2-seropositive hospitalised patients who did not develop an MIS; MIS-P: SARS-CoV-2-seropositive patients with multisystemic inflammatory syndrome; NS: non-significant; SARS-CoV-2: severe acute respiratory syndrome coronavirus 2.

Green: HOS-P; blue: MIS-P; pink: CTL. Antibodies directed against S1, S2, N and full S ectodomain are described for each group of patients. The Kruskal-Wallis ANOVA and Dunn’s multiple comparisons tests were performed for each antigen considered to compare the level of antibody response between the three groups.

### Relationship between SARS-CoV-2 and seasonal HCoV infections

Prevalence rates of anti-S (and to a lesser extent of anti-N) antibodies were generally similar in the CTL, HOS-P and MIS-P groups for the two betacoronaviruses (HKU1 and OC43) and the two alphacoronaviruses (229E and NL63). The exception was the MIS-P group in which the prevalence of HKU1-N, OC43-N and 229E-N antibodies was significantly higher than in the HOS-P or in the CTL groups (Table 3).

**Table 3:** Prevalence of antibodies to seasonal coronaviruses HKU1, OC43, NL63 and 229E spike and nucleoprotein in HOS-P, MIS-P and CTL children, France, April–June 2020 (n =184)

Virus	Antigen	HOS-P SARS-CoV-2-positive n = 54		MIS-P SARS-CoV-2-positive n = 15		CTL SARS-CoV-2-negative n = 115		p (CTL vs HOS-P)	p (CTL vs MIS-P)	p (HOS-P vs MIS-P)
		n	%	n	%	n	%			
HCoV-HKU1	Full S	46	85.2	15	100	100	87.0	NS	NS	NS
	N	19	35.2	11	73.3	52	45.2	NS	NS	0.0166
HCoV-OC43	Full S	50	92.6	15	100	111	96.5	NS	NS	NS
	N	18	33.3	12	80.0	47	40.9	NS	0.0053	0.0024
HCoV-229E	Full S	44	81.5	13	86.7	77	67.0	NS	NS	NS
	N	12	22.2	9	60.0	34	29.6	NS	0.0372	0.0095
HCoV-NL63	Full S	NA	NA	NA	NA	NA	NA	NA	NA	NA
	N	49	90.7	14	93.3	101	87.8	NS	NS	NS

CTL: SARS-CoV-2-seronegative control group; HOS-P: SARS-CoV-2-seropositive hospitalised patients who did not develop an MIS; MIS-P: SARS-CoV-2-seropositive patients with multisystemic inflammatory syndrome; HCoV: seasonal coronaviruses; NA: not applicable; NS: non-significant; SARS-CoV-2: severe acute respiratory syndrome coronavirus 2.

**Figure 2:**
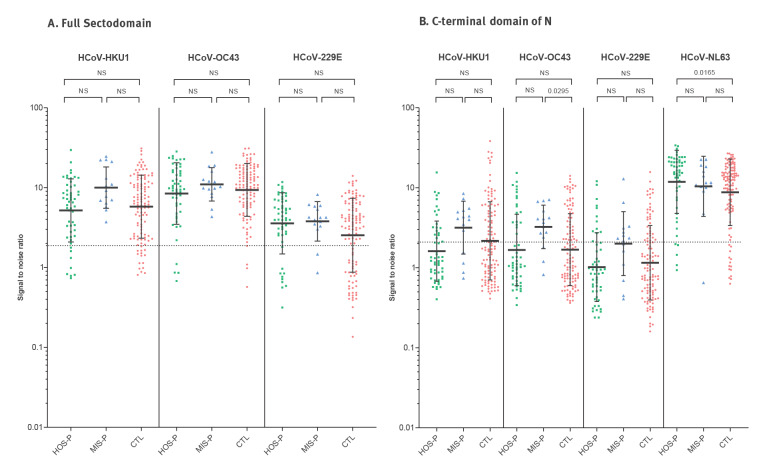
Antibody responses against seasonal HCoV in HOS-P, MIS-P and CTL children, France, April–June 2020 (n =184)

CTL: SARS-CoV-2-seronegative control group; HCoV: human coronavirus; HOS-P: SARS-CoV-2-seropositive hospitalised patients who did not develop an MIS; MIS-P: SARS-CoV-2-seropositive patients with multisystemic inflammatory syndrome; N: nucleoprotein; NS: non-significant; S: spike.

We reasoned that if prior infection with seasonal HCoV induced cross-reactive immunity, this should be reflected in lower SARS-CoV-2 antibody prevalence or lower antibody levels compared with HCoV-naïve patients. Our results did not show any significant difference between HOS-P and CTL patients regarding antibody levels to the four seasonal HCoV (Figure 2).

Green: HOS-P; blue: MIS-P; pink: CTL. Antibodies directed against full S ectodomain and N domain are described for each group of patients. The Kruskal-Wallis ANOVA and Dunn’s multiple comparisons tests were performed for each antigen considered to compare the level of antibody response between the three groups.

**Figure 3:**
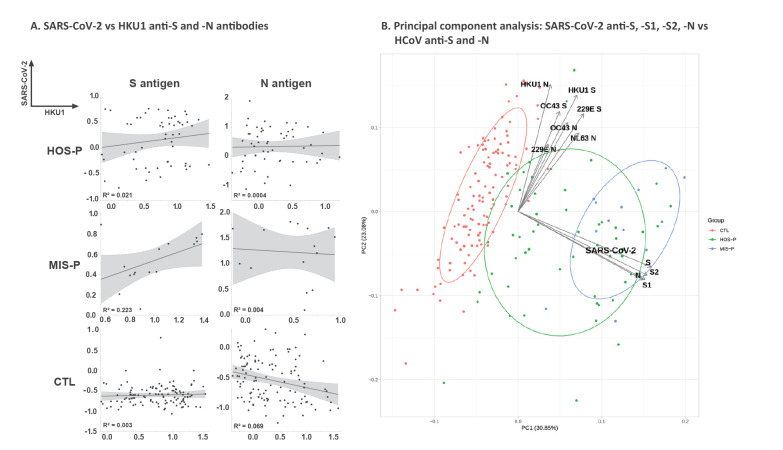
Correlation of SARS-CoV-2 antibody responses with seasonal HCoV, France, April–June 2020 (n =184)

CTL: SARS-CoV-2-seronegative control group; HCoV: human coronavirus; HOS-P: SARS-CoV-2-seropositive hospitalised patients who did not develop an MIS; MIS-P: SARS-CoV-2-seropositive patients with multisystemic inflammatory syndrome; N: nucleoprotein; S: spike; SARS-CoV-2: severe acute respiratory syndrome coronavirus 2.

This observation was confirmed by the PCA analysis, which showed that the patient groups (HOS-P, MIS-P and CTL) were only clustered by SARS-COV-2 antibodies and not by seasonal HCoV antibodies (Figure 3). Therefore, the frequency of SARS-CoV-2 infections and of related MIS diseases were not shaped by prior seasonal HCoV infections. In addition, there was no significant correlation between SARS-CoV-2 and seasonal HCoV antibody levels in HOS-P and MIS-P patients (Supplement, part 3). The level of SARS-CoV-2 antibodies to N and S were correlated, which corresponds to a good internal control. This was also the case for the N and S responses for each HCoV, but to a lesser extent (Supplement, part 3).

The principal component analysis shows that (i) the distribution of all antibodies against SARS-CoV-is 2 mainly correlated with the x-axis and those corresponding to seasonal HCoV with the y-axis and (ii) antibodies against seasonal HCoV do not separate HOS-P and MIS-P from CTL children. This demonstrates the independence of antibody responses between SARS-CoV-2 and HCoV.

## Discussion

Observations indicate that children are less likely to develop COVID-19, and the clinical course of COVID-19 in children is less severe than in adults. To investigate reasons explaining decreased severity of SARS-CoV-2 infection in children, we studied the impact of prior infections with seasonal HCoV on the risk of infection by SARS-CoV-2.

Seasonal HCoV include alphacoronaviruses (229E and NL63) and betacoronaviruses of lineage A (OC43 and HKU1) which primarily replicate in the respiratory tract and mostly cause common colds [28]. These viruses show a worldwide distribution and multiple HCoV infections in various combinations are common [15,28-31]. Infection takes place in very early childhood, children experiencing cough, sore throat, fever and headache. Seroprevalence studies show very high prevalence rates, up to 100% in adult populations [15,32-34]. Our results also showed very high antibody prevalence and therefore we assume that infection by HCoV preceded infection by SARS-CoV-2 in our cohort, although the timing of those prior infections is unknown.

It has been suggested that previous seasonal HCoV infections could impact SARS-CoV-2 replication. We found no evidence of cross-protective immunity linked to previous infection with seasonal HCoV. Firstly, the seasonal HCoV prevalence in SARS-CoV-2-positive and -negative patients was similar. Secondly, there was no significant correlation between SARS-CoV-2 and antibody levels of any HCoV, whatever the antigen considered (S or N), while SARS-CoV-2 antibodies to N and S were correlated as expected.

It must be underlined that antibodies were considered as evidence of past infection by HCoV, the intensity of the antibody response reflecting partly the degree of replication within the host, as an indicator of underlying cellular responses. Indeed, antibodies are unlikely to act as primary effectors of protection, as there is no or very low cross-neutralisation between these coronaviruses [16].

The lack of HCoV/SARS-CoV-2 cross-protection demonstrated here contrasts with the recent identification of pre-existing immune effectors recognising SARS-CoV-2 in healthy subjects sampled before the SARS-CoV-2 pandemic, notably T-helper CD4^+^ cells or IgG antibodies targeting the more conserved antigenic domains including the C-terminal part of S or the nucleoprotein [18,35]. A very sensitive cytometric assay reported frequent low levels of cross-reacting anti-S IgG, mainly targeting the S2 domain of the SARS-CoV-2 spike [16]. However, the clinical relevance of this result is questionable as it is based on a pseudo-neutralisation assay (SARS-CoV-2 pseudotyped lentiviruses expressing SARS-CoV-2 spike protein) in a non-respiratory cellular system (HEK293 cell line) and the mechanism of entry was not physiological because it did not involve the ACE2 receptor of the virus. In this context, our results obtained in a prospective multicentre paediatric study during the first pandemic wave in France are very relevant. They show that infection by endemic seasonal HCoV, and therefore cross-reacting T-cells, do not seem to confer any significant protection against SARS-CoV-2 infection. Importantly, this also suggests that potentially cross-reactive CD8^+^ or CD4^+^ T-cells, which should be elicited upon seasonal HCoV infections as described in SARS-CoV-2 infections [36], do not significantly contribute to protection against SARS-CoV-2 infection. However, our study based on a sample of 184 patients has several limitations as it revealed prior infections by HCoV and infections by SARS-CoV-2 but did not explore underlying mechanisms: (i) the T-CD4^+^ and T-CD8^+^ cellular responses were not studied, (ii) despite providing evidence of past HCoV infection, the delay between HCoV infection and SARS-CoV-2 infection was unknown and (iii) the quality of the HCoV antibodies in terms of, for instance, their neutralisation potential was not known.

As common colds and mild bronchitis caused by seasonal HCoV are experienced repeatedly, we need to question whether coronavirus infections induce a long-term clinically protective immune response based on antibody responses or on other immune effectors. Indeed, a recent study showed that protective immunity against the four coronaviruses was short-lasting [32]. Our results therefore cast doubt on whether the systemic humoral response against SARS-CoV-2 can be a good indicator of herd immunity, even if the prevalence of antibodies becomes high in the population.

Multisystem inflammatory syndrome is exceptionally rare, around 25 per 100,000 children younger than 5 years in North America [25]. Viral respiratory agents, including seasonal coronaviruses, have been reported as triggers for MIS [37]. In the Paris area, a 13-fold increased incidence in MIS was reported during the first COVID-19 pandemic wave compared with the 2 previous years [23], evidenced a temporal association and strongly suggested a causal link between MIS and SARS-CoV-2 infection. We therefore analysed the SARS-CoV-2 antibody profile in MIS cases. We found higher antibody concentrations against S1 and N than in HOS-P patients who experienced an asymptomatic or pauci-symptomatic infection. This was not the case for serum antibody concentrations against beta- (OC43) or alpha- (229E and NL63) coronaviruses, suggesting that this increased response is specific to SARS-CoV-2 infection. Furthermore, it has been suggested that the lack of cross-reactivity between anti-S1 antibodies of the different seasonal and SARS-CoV-2 viruses does not favour the hypothesis of SARS-CoV-2 infection boosting pre-existing HCoV immunity in MIS patients [35]. As a whole, our data do not support that previous HCoV infection facilitates SARS-CoV-2 infection and MIS-related disease. The anti-N (but not anti-S) prevalence in HKU1, OC43 and 229E infections were higher in MIS-P patients than in CTL and HOS-P patients. This was not associated with differences in quantitative anti-N or anti-S antibody responses. Higher N antibody frequency in MIS-P may have originated from back-boosting cross-reactivity for HCoV N-specific responses as epitopes are shared between HCoV and SARS-CoV2 [38], and anamnestic responses to HCoV may influence the antibody response to SARS-CoV-2 [39]. Isotype or avidity of these responses was not tested here but could help to assess functional maturation of N-specific antibodies in future studies. In addition, we acknowledge that we could have failed to identify a succession of events, for example the time period between a first infection with an HCoV and the SARS-CoV-2 infection, which may have led to a specific pattern of antibodies and contributed to MIS physiopathology.

Most of the patients reported no symptoms suggestive of acute COVID-19 disease and most had positive serum IgG responses. This suggests that the development of MIS in these patients probably was the result of a post-viral immunological reaction. It should also be stressed that other causes than COVID-19 can be responsible for MIS. During the first wave of the outbreak (26 April to 26 May), 30 children with MIS were admitted in our hospital, among whom 23 had a positive SARS-CoV-2 serology and seven were negative for both SARS-CoV-2 PCR and serology [40].

Our results also show that children present with a very high antibody prevalence to seasonal HCoV, which does not impair the efficient circulation of these viruses every year, pointing to the limits of herd immunity applied to seasonal coronaviruses, and possibly SARS-CoV-2.

## Supplementary Data

1566000 Supplement
