# Supplementary material for: Prior infection by seasonal coronaviruses, as assessed by serology, does not prevent SARS-CoV-2 infection and disease in children, France, April to June 2020
Source: Euro Surveill. 2021 Apr 1;26(13):2001782. doi: 10.2807/1560-7917.ES.2021.26.13.2001782 (PMC8017906; doi:10.2807/1560-7917.ES.2021.26.13.2001782)
Supplement: Supplement [file 20-01782_ELOIT_supplement.pdf]

Supplementary material

This supplementary material is hosted by *Eurosurveillance* as supporting information alongside the article *Prior infection by seasonal coronaviruses, as assessed by serology, does not prevent SARS-CoV-2 infection and disease in children, France, April to June 2020*, on behalf of the authors, who remain responsible for the accuracy and appropriateness of the content. The same standards for ethics, copyright, attributions and permissions as for the article apply. Supplements are not edited by *Eurosurveillance* and the journal is not responsible for the maintenance of any links or email addresses provided therein

Table of contents

1-Serological methods: description of LIPS assay 2

2- Calculation of Se and Spe for Ab tests for SARS-CoV-2 (N, S1, S2, S) and seasonal HCoVs 3

2.1 Sensitivity and specificity of SARS-CoV-2 Ab tests 3

2.2 Calculation of Se and Sp for Ab tests for seasonal HCoVs 4

2.2.1 Preliminary remark 4

2.2.2 Background and methods 4

2.2.3 Results 6

3- Correlation between variables 8

4-References 11

1-Serological methods: description of LIPS assay

All SARS-CoV-2 antigens were previously described in Grzelak et al[1] Briefly, synthetic genes coding for the S1 (residues 1-698) and S2 (residues 686-1208) subdomains of the spike protein, along with the full S ectodomain (residues 1-1208) and the carboxy-terminal part (residues 233-419) of the nucleoprotein were ordered from GenScript with codon usage optimized for protein expression in mammalian cells, and cloned in the pcDNA3.1(+) vector. An exogenous signal peptide was added to S2 to ensure efficient protein secretion. The S ectodomain construct was engineered as reported before[2] to have the stabilizing double proline mutation ($KV^{986\text{-}987}$ to $PP^{986\text{-}987}$) and the foldon domain at the C-terminus[3] that allows the S to trimerize (YIPEAPRDGQAYVRKDGEWVLLSTFL), resembling the native S state on the virion. The nanoluc was directly added to the carboxy-terminal end of the constructs (N construct), or spaced by a 3-residues linker (S-based constructs).

For seasonal coronaviruses, similar designs were performed. The carboxy-terminal parts of the nucleoproteins were identified by aligning complete N protein sequences of HCoVs with the SARS-CoV-2 N protein sequence. The spike ectodomain of HCoV-OC43 (residues 15-1263) was expressed using the exogenous CD5 signal peptide (MPMGSLQPLATLYLLGMLVASVL) as reported[1], with the GCN4 trimerization domain (IEDKIEEIESKQKKIENEIARIKKIK) appended to the ectodomain C-terminus, followed by a GSG linker and the nanoluc. The ectodomain had a mutated furin site to increase protein stability (754-RRSRG-758 → 754-GGSGG-758). The HCoV-HKU1 construct contained an exogenous signal peptide derived from a mouse leader sequence for Ig kappa chain (METDTLLLWVLLLWVPGSTG) added at the N-terminus of the ectodomain (residues 14-1276). The furin site was mutated (752-RRKRR-756 → 752-GGSGS-756) as previously reported[2]. Residues 1067 and 1068 (NL) were mutated to PP to increase protein yields, as already described[4]. T4 fibritin trimerization domain (YIPEAPRDGQAYVRKDGEWVLLSTFL) was finally added to the C-terminus of the ectodomain, followed by the GSG linker and by the nanoluc. The HCoV-229E recombinant spike protein was constructed with the same signal peptide as the HCoV-HKU1 construct, followed by the ectodomain (residues 17-1113), and the T4 fibritin-GGS-nanoluc C-terminal extension. Residues 871 and 872 (TI) were mutated to PP to stabilize the pre-fusion state of the protein, as reported[4].

Recombinant proteins were produced and quantified as previously described[5].

We used as first line assay the test we previously implemented[5]. The specificity (Spe) of the LIPS assay for each antigen (carboxy-terminal part of N [hereafter named N], S1 and S2) was > 98.5% (Figure S1). We considered that a serum was positive if antibodies (Ab) to at least one of the three antigens were detected, which provided a Spe > 97%. With this algorithm, the sensitivity (Se) analysed in a population of 183 individuals in a cluster of already reported mild or asymptomatic cases sampled one to two months after the index case was 88% (Table S1). Use of full S as antigen (Ag) was more sensitive (99.4%) but as reported by others[8] some pre-epidemic sera showed a strong reactivity (main text, Figure 1) and we decided not to use it as a first line test.

Similarly to the protocol for SARS-CoV-2[5], we extended LIPS tests to seasonal coronaviruses and designed assays targeting the carboxy-terminal part of N of the four seasonal HCoVs, and the trimeric ectodomain of spike in a prefusion state of three out of the fur coronaviruses (except NL63 which is an alphacoronavirus). Because most of the population has been exposed to seasonal HCoVs, defining a cut-off was challenging. As the LIPS assays were strictly identical for the five viruses, including the mode of preparation of antigens and their molecular concentration, thanks to a quantification based on nanoluciferase fused to each antigen, we tentatively used for seasonal HCoVs the same cut-off as for SARS-CoV-2 for each antigen, using pre-epidemic sera (Figure S1). Based on this cut-off, we then confirmed that the apparent prevalence of seasonal HCoVs in very young children (1-2 years old) was lower than in adults, as expected (Figure S1). This was an indirect evidence of the specificity of the test. We then integrated the results of these two populations with different prevalences to estimate the Se and Spe of seasonal HCoV tests.

2- Calculation of Se and Spe for Ab tests for SARS-CoV-2 (N, S1, S2, S) and seasonal HCoVs

2.1 Sensitivity and specificity of SARS-CoV-2 Ab tests

For establishing the specificity of the tests used in first line, we used 48 sera belonging to a collection of 260 sera of pre-epidemic healthy donors (230 sera from the Diagmicoll cohort collection of the ICAReB platform[6] approved by CPP Ile-de-France I, sampled before November 2019 (Grzelak et al.); and 40 sera from children 12-24 month (kindly provided by Julie Toubiana). The ICAReB platform (BRIF code n°BB-0033-00062) of Institut Pasteur collects and manages bioresources following ISO 9001 and NF S 96-900 quality standards**.** Due to limits of volume available for children, SARS-CoV-2 full S, S2 and N specificity were evaluated with 30 sera and the specificity of S1 with the other 10 sera (Figure S1). All the S1, S2 and N responses were below the cut-off, which defines a specificity of 100% (lower bound: 98.5 % [binomial calculation, one-sided 97.5% confidence interval]). The lower bound of the specificity for the algorithm used to include the patients (positivity either for S1, S2, or N) is therefore > 0.985^3 = >95.6%.
For establishing the sensitivity of the tests used in first line, sera already deeply analyzed with a set of techniques from a COVID-19 cluster in France[5,7] were used. This study was registered with ClinicalTrials.gov (NCT04325646) and received ethical approval by the Comité de Protection des Personnes Ile de France. Informed consent was obtained from all participants. Serum samples were taken from individuals with mild symptoms and contacts, to mimic the HOS population of children to be tested (avoiding selecting hospitalized patients that generally develop higher titers of antibodies that are easier to detect). We selected all 183 samples that were found positive with the S-Flow assay, which is based on the recognition of S at the cell surface by flow-cytometry and was demonstrated to be highly sensitive[8].

Figure S1: Distribution of antibody responses to SARS-CoV-2 and seasonal HCoVs in pre-epidemic sera from adults and infants. Blue lines represents the positivity threshold for each assay.

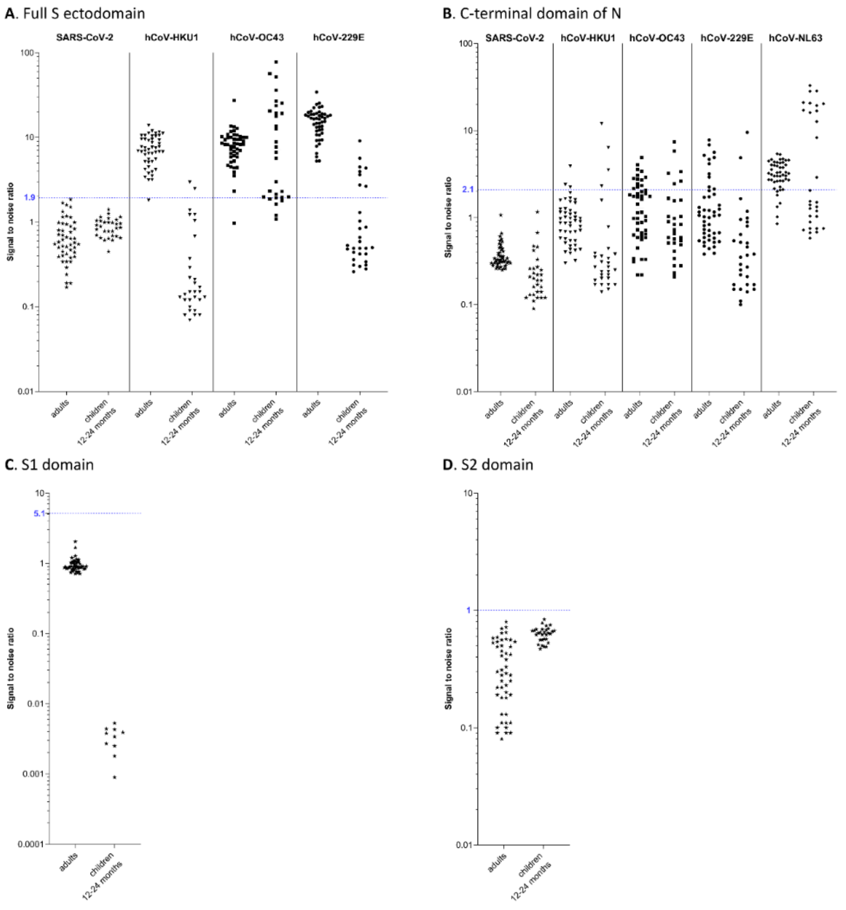

Table S1: **Sensitivity of LIPS targeting different antigens: number of positive sera among 183 S-Flow positive sera**. The sensitivity (Se) for each antigen and of the algorithm used to include the patients (N or S1 or S2 positive) is depicted. The sensitivity of detection of Abs to N and S, used to compare prevalence rates with those of seasonal HCoVs is also shown [confidence interval, p=0.05].

	S-Flow positive sera (n=183)	
	No of positive sera	% of positive sera
LIPS S	182	99.4 [97.0-100]
LIPS N	152	83.1 [76.8-88.1]
LIPS S1	97	53.3 [45.5-60.4]
LIPS S2	146	79.8 [73.2-85.3]
LIPS N or S1 or S2 (used as first line to define SARS-CoV-2 serological status of included children)	161	88.0 [82.4-92.3]

2.2 Calculation of Se and Sp for Ab tests for seasonal HCoVs

2.2.1 Preliminary remark

To evaluate the prevalence of prior infections with the four seasonal HCoVs we have developed Ab tests based exactly on the same methodology as for SARS-CoV-2, allowing for unbiased comparison. We have evaluated the sensitivity and specificity of these HCoVs antibody tests, using the approach described below. Readers not interested by the statistical approach can access directly to the Table S6 that shows Se and Sp values.

2.2.2 Background and methods

Unlike SARS-CoV-2, the four human coronaviruses (HKU1, NL63, OC43, and 229E) are endemic worldwide. Their apparent seroprevalences are above 70% in adults[9,10] and seroconversion mainly occurs during childhood before the age of six[10]. To clarify the true seroprevalence in populations, assessment of the sensitivity and specificity of serological tests against HCoVs is required. However, without any known Gold standard test (*i.e.* a test with a sensitivity and specificity equal to 100%), the sensitivity and specificity cannot be assessed by usual approaches. In this context, latent class models, as developed by Hui and Walter in 1980[11], have proven their usefulness to assess the sensitivities and specificities of multiple diagnostic tests without knowing the true status of the tested individuals[12,13]. They rely on modelling of the crossed results from two or more tests performed in at least two populations with distinct prevalences. The objective was to assess the sensitivities and specificities of both LIPS S and LIPS N assays for seasonal HCoVs performed in the two pre-epidemic cohorts of the ICAReB platform[6] with 48 sera from adults, for whom a high seroprevalence against endemic HCoV is expected, and 30 sera from children 12 to 24 months old, for whom a low seroprevalence against endemic HCoVs is expected. Results obtained from the SARS-CoV-2 LIPS S and LIPS N assays were also included in the analysis as an additional HCoV, although the sensitivities related to these assays could not be assessed based on these pre-epidemic sera.

Since the individual immunologic response and kinetics of antibodies directed against HCoVs may vary from one coronavirus to another, we assumed that the sensitivities of both LIPS N and LIPS S may vary as well. In contrast, false positive results mainly occur due to cross reactivity of antibodies and, as we identically designed and performed all tests for every HCoV, we assumed that false positive results occurred with the same frequency for all HCoVs. Thus, specificities of the LIPS S and LIPS N assays for each HCoV were assumed to be equal, including the one for SARS-CoV-2.

Although the assays target specific antibodies (toward N or full S antigens), both assays rely on the same biological mechanisms. For this reason, we assumed that the LIPS S and LIPS N assays are conditionally dependent of the true serological status[12]. To assess the sensitivities and specificities of both LIPS S and LIPS N assays targeting the four HCoVs, we adapted the "two tests - two populations" model introduced by Vacek[14] which considered the conditional dependence between tests with a fixed effect for truly seropositive and for truly seronegative individual[15,16].

The directed acyclic graph of the model is presented in Figure S2.

Figure S2: Directed Acyclic Graph of the latent class model. Every nod is, if necessary, indexed by the population number ($j \in \left[1; N_{pop}\right]$) and HCoV number ($i \in \left[1; N_{HCOV}\right]$). Plain arrows represent stochastic links and dotted represent deterministic links. Vectors are indicated with two square brackets. Measured variable (grey oval) is n_{ij}, a vector of four dimensions which correspond to the number of individuals in each of the four combinations of the two-test results (LIPS N and LIPS S) for the i th HCoV in the j th population. Unknown parameters (white ovals) include sensitivities of the two tests for each HCoV and specificities for the two tests, conditional dependence terms.

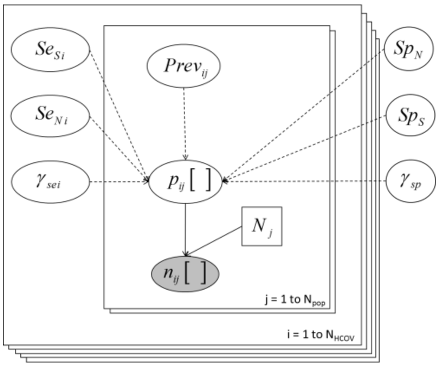

Statistical model

We noted n_{ij} the vector of length 4 corresponding to the number of individuals in each of the four combinations of the two tests results of the ith HCOV in the jth cohort. It follows a multinomial distribution of size N_j (the number of sera in the jth cohort) and of probability vector p_{ij} (Table S2).

$$n_{ij} \sim multinomiale\left(N_{ij}, p_{ij}\right)$$

To ensure that all conditional probabilities (of Table S2) are included in the $[0;1]$ interval, we implemented the inequality constraints as proposed by Wang et al.[17] in JAGS[18]. We also constrained the sensitivities to be superior to the complement of the respective specificities ($Se \in [1-Sp;1]$) to avoid unidentifiability related to the existence of a mirror image with the same likelihood (when the model switches labels of truly positive and truly negative)[19].

Table S3: Probability vector of the multinomial distribution of n_{ij}. We identify each of the 4 combinations of test results by the results of each test (for example, the $LIPS_S^- LIPS_N^-$ category corresponds to individuals negative to LIPS S and LIPS N).

$p_{ij}[\ ]$	**Combination**	**Probability**
$p_{ij}[1]$	$LIPS_S^- LIPS_N^-$	$Prev_{ij} \times \left((1-Se_{Si}) \times (1-Se_{Ni}) + \gamma_{Sei}\right) + \left(1-Prev_{ij}\right) \times \left(Sp_S \times Sp_N + \gamma_{Sp}\right)$
$p_{ij}[2]$	$LIPS_S^- LIPS_N^+$	$Prev_{ij} \times \left((1-Se_{Si}) \times Se_{Ni} - \gamma_{Sei}\right) + \left(1-Prev_{ij}\right) \times \left(Sp_S \times (1-Sp_N) - \gamma_{Sp}\right)$
$p_{ij}[3]$	$LIPS_S^+ LIPS_N^-$	$Prev_{ij} \times \left(Se_{Si} \times (1-Se_{Ni}) - \gamma_{Sei}\right) + \left(1-Prev_{ij}\right) \times \left((1-Sp_S) \times Sp_N - \gamma_{Sp}\right)$
$p_{ij}[4]$	$LIPS_S^+ LIPS_N^+$	$Prev_{ij} \times \left((1-Se_{Si}) \times (1-Se_{Ni}) + \gamma_{Sei}\right) + \left(1-Prev_{ji}\right) \times \left(Sp_S \times Sp_N + \gamma_{Sp}\right)$

Where $Prev_{ij}$ is the seroprevalence of the ith HCoV in the jth cohort, Sp_S and Sp_N are the specificities of the LIPS S and LIPS N assays common to all HCoVs, Se_{Si} and Se_{Ni} are respectively the sensitivities of the LIPS S and LIPS N assays for the ith HCoV, and γ_{Sei} and γ_{Sp} are respectively the conditional dependence terms for truly seropositive sera of the ith HCoV and conditional dependence term for truly seronegative sera.

Note that, LIPS S was not available for HCoV NL63 and only the results of LIPS N were modelled. So in the jth cohort, n_{NL63j}, the number of positive sera to the LIPS N for HCoV NL63, was supposed to follow a binomial distribution of size N_j and of probability $p_j = Prev_{NL63j} \times Se_{N\,NL63} + \left(1 - Prev_{NL63j}\right) \times \left(1 - Sp_N\right)$.

Prior Distributions

Except $P_{jSARSCOV2}$ which was fixed to 0 because the two cohorts were sampled before November 2019, vaguely informative priors (Table S3) were assigned to each parameter allowing variation within a realistic range and forcing probability parameters ($Se_{\bullet}$, $Sp_{\bullet}$ and $Prev_{\bullet\bullet}$) to be comprised between 0 and 1. We used a Cauchy distribution, with 2.5 and 97.5 percentiles respectively fixed at -0.5 and 0.5, as prior for all conditional dependence terms in the model. This prior supported posterior estimates with relatively low conditional dependence values (since the Cauchy distribution is centred and relatively picked at 0) while also allowing the model to converge toward other posteriors when conditional terms were relatively high.

Table S4: Prior distribution of unknown parameters

Nodes	Prior
$Prev_{ij}$	$\text{beta}\left(0.5, 0.5\right)$
$Se_{S\,HCOVi}, Se_{N\,HCOVi}, Sp_S, Sp_N$	$\text{beta}\left(0.5, 0.5\right)$
$\gamma_{SeHCOVi}$ γ_{Sp}	$\text{dcauchy}\left(0.039, 0.039\right)$

Monte Carlo Markov Chain (MCMC) techniques were used to estimate the full joint posterior distribution of parameters from prior distributions and data. Computations were performed using the JAGS software via the R package rjags[18] (a corresponding R script is available on request). We ran three independent MCMC chains in parallel and for each chain 110,000 samples were produced. The first 10,000 were discarded as burn-in; the remaining 100,000 samples were thinned by selecting one out of 20 samples to cope with autocorrelation, thus keeping 5,000 samples per chain. The convergence was checked again by displaying MCMC chain traces and autocorrelation plots and by computing the Gelman and Rubin's statistics as modified by Brooks and Gelman[20]. For each parameter, point estimate was defined as the median of its marginal posterior distribution, and the 95% credible interval was defined by the 2.5 and 97.5 percentiles of this marginal distribution.

2.2.3 Results

To maximize the specificity of SARS-CoV-2 assays, we fixed the respective cut-off of LIPS S and LIPS N assays to 1.9 and 2.1[5] for all HCoVs. Crossed test results of LIPS S and LIPS N for each HCoV are displayed in Table S4. The point estimates and 95% credibility interval of every parameter are displayed in Table S5.

Table S5: Crossed tests results of LIPS S and LIPS N for all tested HCoVs.

HCoV	cohort	$LIPS_S^- LIPS_N^-$	$LIPS_S^- LIPS_N^+$	$LIPS_S^+ LIPS_N^-$	$LIPS_S^+ LIPS_N^+$
SARS-CoV-2	Adults	48	0	0	0
	Children	30	0	0	0
HKU1	Adults	1	0	44	3
	Children	26	2	0	2
OC43	Adults	1	0	34	13
	Children	5	0	19	6
229E	Adults	0	0	37	11
	Children	21	1	7	1
		$LIPS_N^-$		$LIPS_N^+$	
NL63	Adults	7		41	
	Children	17		13	

Table S6: Estimations of each parameter point estimates and 95% credibility intervals (in square brackets) are respectively the median and the 2.5 and 97.5 percentiles of the posterior distribution. The seroprevalence of SARS-CoV-2 in adults and children are fixed to 0, so sensitivities of LIPS assays for SARS-CoV-2 cannot be estimated by this approach (see Table S2).

Parameters	**HCoV**				
	SARS-CoV-2	HKU1	OC43	229E	NL63
P_{adult}	0	0.989 [0.922;1]	0.989 [0.923;1]	0.995 [0.949;1]	0.929 [0.766;1]
$P_{children}$	0	0.016 [0;0.142]	0.853 [0.681;0.981]	0.266 [0.125;0.45]	0.453 [0.257;0.689]
Se_S	NA	0.967 [0.89;0.996]	0.969 [0.893;0.997]	0.977 [0.914;0.998]	NA
Se_N	NA	0.091 [0.04;0.19]	0.264 [0.173;0.37]	0.222 [0.128;0.343]	0.917 [0.763;1]
$\gamma_{SeHCOVi}$	NA	-0.006 [-0.039;0.004]	0 [-0.02;0.018]	-0.004 [-0.033;0.007]	NA
Common parameters for all HCoV (including SARS-CoV-2)					
Sp_S	0.979 [0.945;0.996]				
Sp_N	0.963 [0.926;0.987]				
γ_{Sp}	0.012 [0.001;0.035]				

3- Correlation between variables

Figure S3 shows the absence of correlation between SARS-CoV-2 and seasonal HCoV level of Abs in HOS-P and MIS-P patients. The level of SARS-CoV-2 antibodies to N and S were correlated, which corresponds to a good internal control. This was also the case for the N and S responses for each HCoV, but to a lesser extent (Supplementary Figure 3). We hypothesize that this might be due to multiple infections by seasonal HCoVs which would boost Ab responses against shared epitopes that are more frequent in the nucleoprotein than in the spike, and that this would lead to a decrease of correlation between N and S responses over time.

The same level of correlation for the HCoVs-S and -N responses was observed in SARS-CoV-2 seronegative CTL patients. This was further confirmed by the Principal Component Analysis (Figure S4). Those results provide evidence that infection with SARS-CoV-2 does not significantly boost pre-existing antibody responses to HCoVs N and S (Figure S3).

Figure S3: Correlation of antibodies against seasonal HCoVs Spike to antibody against Nucleoprotein in each category of SARS-CoV-2 status

A) HOS-P SARS-CoV-2 positive children B) MIS-P SARS-CoV-2 positive children C) CTL SARS-CoV-2 negative children. Horizontal and vertical labels correspond respectively to the level of AB against SARS-CoV2 S1, S2, N and full S; HKU1 S and N; OC43 N and S; 229 E N and S; NL63N. The correlation coefficient for each combination is also shown. In the diagonal are shown the density plots for the variable.

A) HOS-P SARS-CoV-2 positive

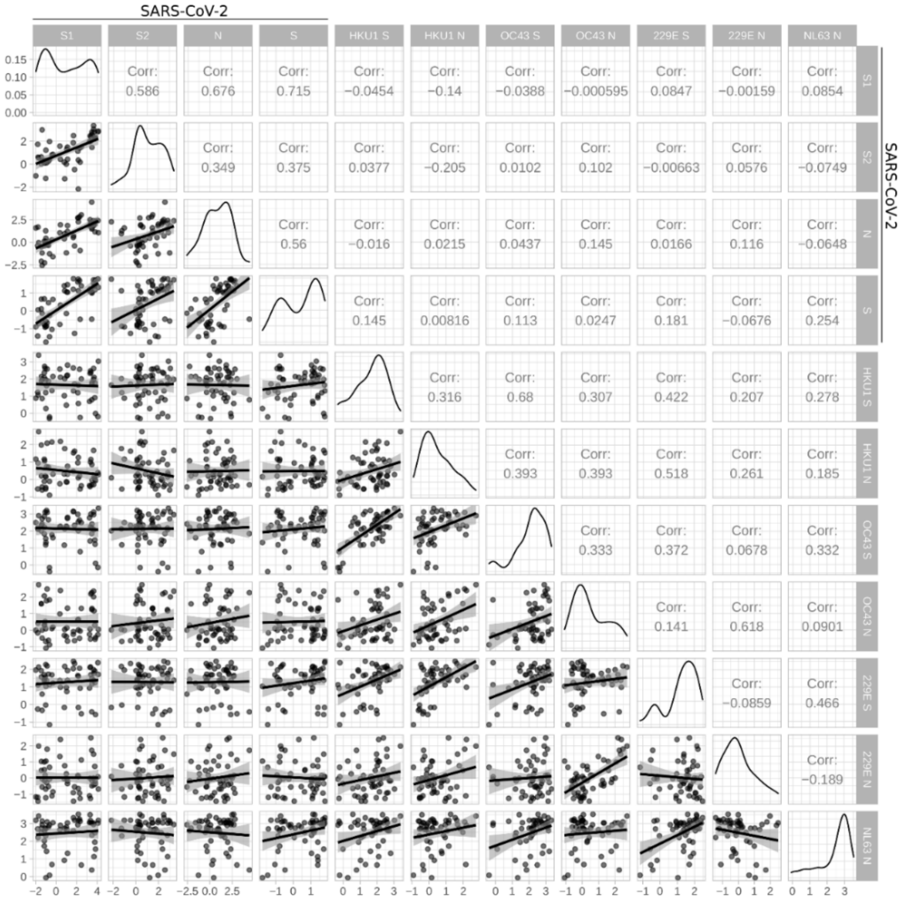

B) MIS-P SARS-CoV-2 positive

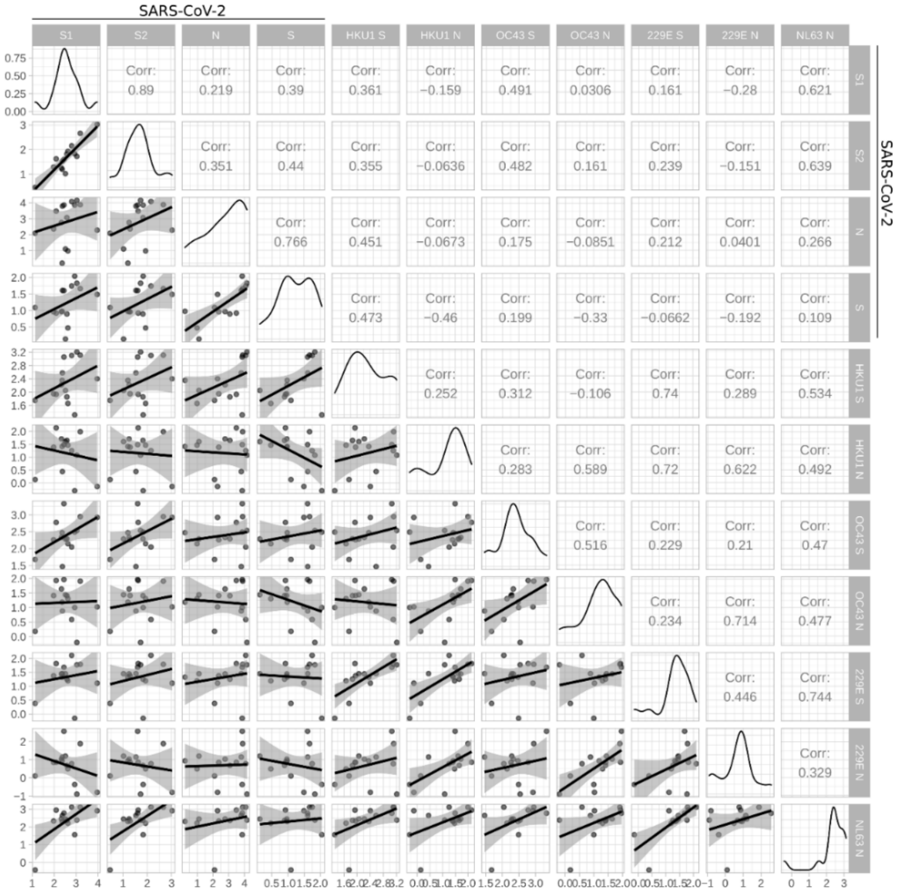

C) CTL

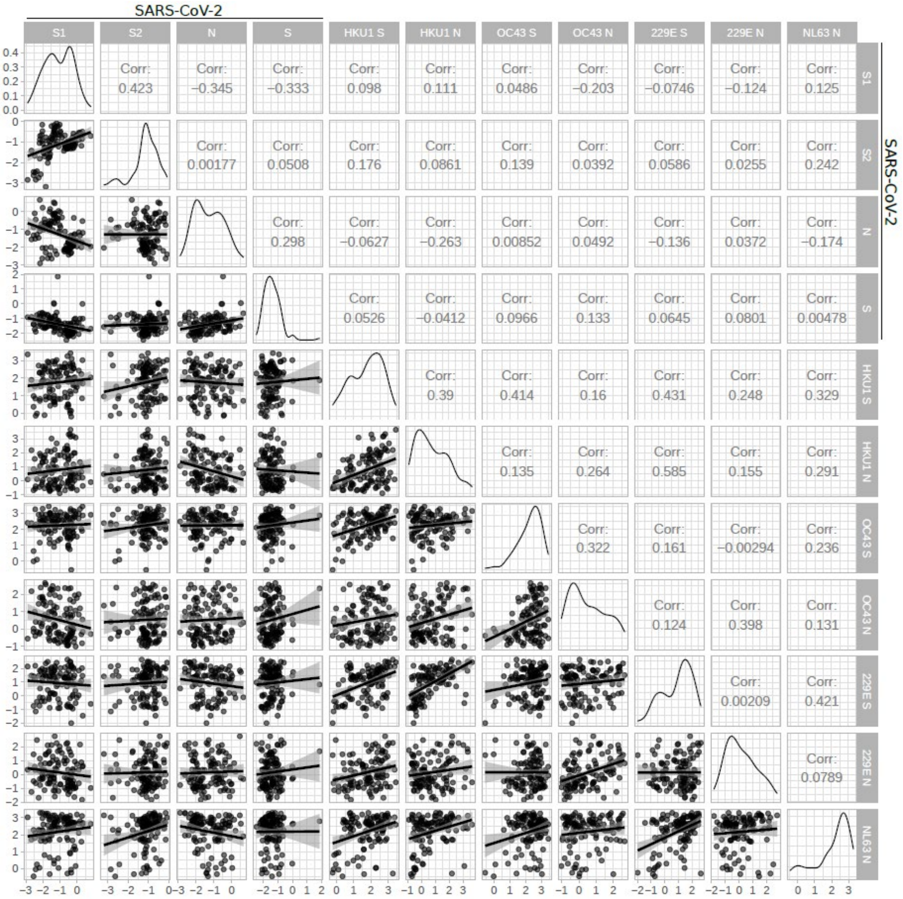

Figure S4: Principal Components Analysis of anti-S and anti-N seasonal HCoV-2 Ab responses in HOS-P, MIS-P and SARS-CoV-2 negative CTL individuals. Because the NL63 Spike assay was not available, the NL63 alphacoronavirus S and N Abs were not included in the analysis

A: Anti-S

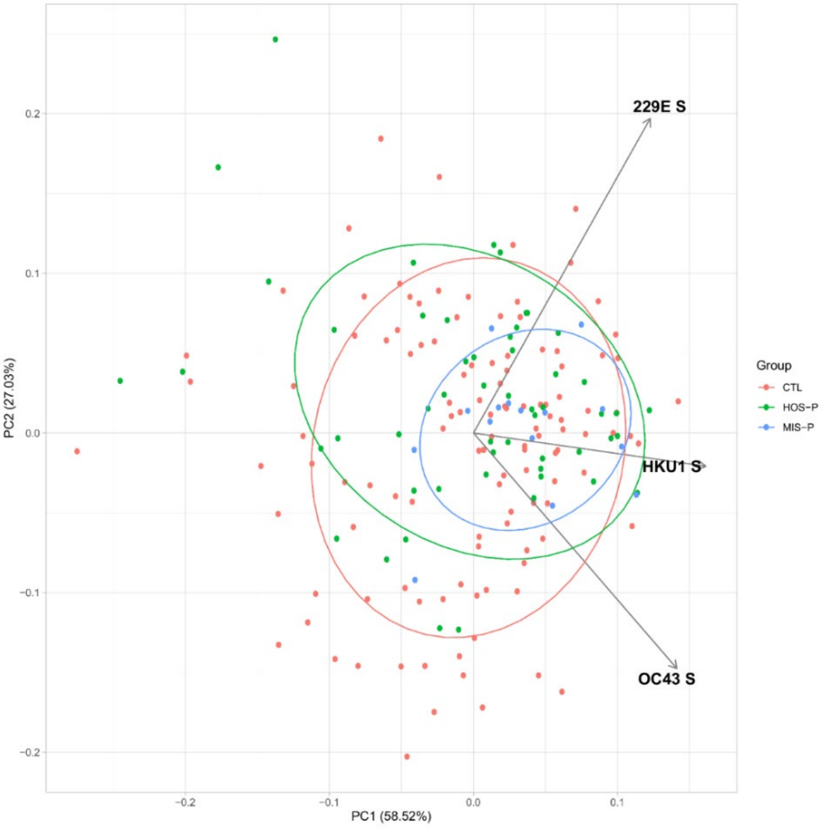

B: Anti-N

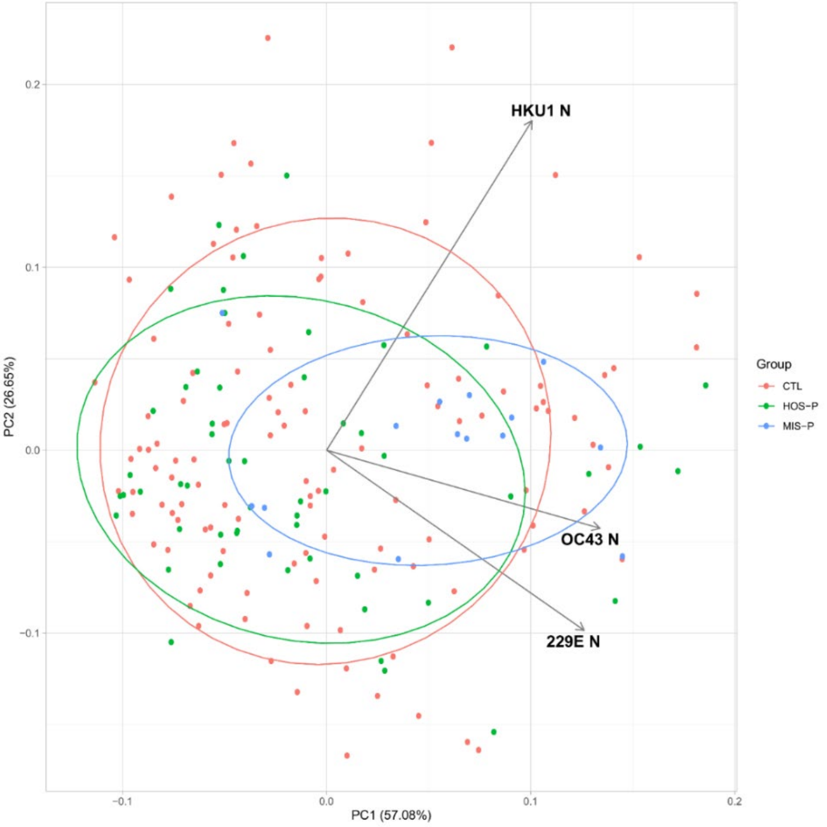

4-References

1 Grzelak L, Temmam S, Planchais C, *et al.* A comparison of four serological assays for detecting anti-SARS-CoV-2 antibodies in human serum samples from different populations. *Science Translational Medicine* 2020; **12**. DOI:10.1126/scitranslmed.abc3103.

2 Kirchdoerfer RN, Wang N, Pallesen J, *et al.* Stabilized coronavirus spikes are resistant to conformational changes induced by receptor recognition or proteolysis. *Scientific Reports* 2018; **8**: 15701.

3 Stevens J, Corper AL, Basler CF, Taubenberger JK, Palese P, Wilson IA. Structure of the uncleaved human H1 hemagglutinin from the extinct 1918 influenza virus. *Science* 2004; **303**: 1866–70.

4 Pallesen J, Wang N, Corbett KS, *et al.* Immunogenicity and structures of a rationally designed prefusion MERS-CoV spike antigen. *Proc Natl Acad Sci USA* 2017; **114**: E7348–57.

5 Grzelak L, Temmam S, Planchais C, *et al.* SARS-CoV-2 serological analysis of COVID-19 hospitalized patients, pauci-symptomatic individuals and blood donors. *medRxiv* 2020; : 2020.04.21.20068858.

6 Esterre P, Ait-Saadi A, Arowas L, *et al.* The ICAReB Platform: A Human Biobank for the *Institut Pasteur* and Beyond. *Open Journal of Bioresources* 2020; **7**: 1.

7 Fontanet A, Tondeur L, Madec Y, *et al.* Cluster of COVID-19 in northern France: A retrospective closed cohort study. *medRxiv* 2020; : 2020.04.18.20071134.

8 Fafi-Kremer S, Bruel T, Madec Y, *et al.* Serologic responses to SARS-CoV-2 infection among hospital staff with mild disease in eastern France. *medRxiv* 2020; : 2020.05.19.20101832.

9 Severance EG, Bossis I, Dickerson FB, *et al.* Development of a Nucleocapsid-Based Human Coronavirus Immunoassay and Estimates of Individuals Exposed to Coronavirus in a U.S. Metropolitan Population. *CVI* 2008; **15**: 1805–10.

10 Zhou W, Wang W, Wang H, Lu R, Tan W. First infection by all four non-severe acute respiratory syndrome human coronaviruses takes place during childhood. *BMC Infectious Diseases* 2013; **13**: 433.

11 Hui SL, Walter SD. Estimating the error rates of diagnostic tests. *Biometrics* 1980; **36**: 167–71.

12 Collins J, Huynh M. Estimation of diagnostic test accuracy without full verification: a review of latent class methods. *Statistics in Medicine* 2014; **33**: 4141–69.

13 Umemneku Chikere CM, Wilson K, Graziadio S, Vale L, Allen AJ. Diagnostic test evaluation methodology: A systematic review of methods employed to evaluate diagnostic tests in the absence of gold standard – An update. *PLoS One* 2019; **14**. DOI:10.1371/journal.pone.0223832.

14 Vacek PM. The effect of conditional dependence on the evaluation of diagnostic tests. *Biometrics* 1985; **41**: 959–68.

15 Branscum AJ, Gardner IA, Johnson WO. Estimation of diagnostic-test sensitivity and specificity through Bayesian modeling. *Preventive Veterinary Medicine* 2005; **68**: 145–63.

16 Georgiadis MP, Johnson WO, Gardner IA, Singh R. Correlation-adjusted estimation of sensitivity and specificity of two diagnostic tests. *Journal of the Royal Statistical Society: Series C (Applied Statistics)* 2003; **52**: 63–76.

17 Wang Z, Dendukuri N, Zar HJ, Joseph L. Modeling conditional dependence among multiple diagnostic tests. *Stat Med* 2017; **36**: 4843–59.

18 Plummer M, Stukalov A, Denwood M. rjags: Bayesian graphical models using MCMC. 2016 https://cran.r-project.org/web/packages/rjags/index.html (accessed June 26, 2016).

19 Engel B, Swildens B, Stegeman A, Buist W, De Jong M. Estimation of sensitivity and specificity of three conditionally dependent diagnostic tests in the absence of a gold standard. *JABES* 2006; **11**: 360.

20 Brooks SP, Gelman A. General methods for monitoring convergence of iterative simulations. *Journal of Computational and Graphical Statistics* 1998; **7**: 434–55.
